# Dedicated cardiac rehabilitation wearable sensor and its clinical potential

**DOI:** 10.1371/journal.pone.0187108

**Published:** 2017-10-31

**Authors:** Hooseok Lee, Heewon Chung, Hoon Ko, Changwon Jeong, Se-Eung Noh, Chul Kim, Jinseok Lee

**Affiliations:** 1 Department of Biomedical Engineering, Wonkwang University College of Medicine, Iksan, Republic of Korea; 2 Department of Rehabilitation Medicine, Wonkwang University Colledge of Medicine, Iksan, Republic of Korea; 3 Department of Rehabilitation Medicine, Sanggye Paik Hospital, Inje University Medical College, Seoul, Republic of Korea; University of Illinois at Urbana-Champaign, UNITED STATES

## Abstract

We describe a wearable sensor developed for cardiac rehabilitation (CR) exercise. To effectively guide CR exercise, the dedicated CR wearable sensor (DCRW) automatically recommends the exercise intensity to the patient by comparing heart rate (HR) measured in real time with a predefined target heart rate zone (THZ) during exercise. The CR exercise includes three periods: pre-exercise, exercise with intensity guidance, and post-exercise. In the pre-exercise period, information such as THZ, exercise type, exercise stage order, and duration of each stage are set up through a smartphone application we developed for iPhones and Android devices. The set-up information is transmitted to the DCRW via Bluetooth communication. In the period of exercise with intensity guidance, the DCRW continuously estimates HR using a reflected pulse signal in the wrist. To achieve accurate HR measurements, we used multichannel photo sensors and increased the chances of acquiring a clean signal. Subsequently, we used singular value decomposition (SVD) for de-noising. For the median and variance of RMSEs in the measured HRs, our proposed method with DCRW provided lower values than those from a single channel-based method and template-based multiple-channel method for the entire exercise stage. In the post-exercise period, the DCRW transmits all the measured HR data to the smartphone application via Bluetooth communication, and the patient can monitor his/her own exercise history.

## Introduction

Cardiovascular disease (CVD) remains the number one cause of death globally. In the US, it was reported that the number of adults with diagnosed heart disease was 28.4 million in 2015, which was 11.7% of the population [1]. The World Health Organization (WHO) has also reported that an estimated 17.5 million people die every year due to CVD, representing 31% of all global deaths [2]. The American Heart Association (AHA) has suggested that active participation in cardiac rehabilitation (CR) exercise after cardiac disease is effective in lowering the recurrence rate of cardiac disease, indicating the importance of engaging in CR exercise [3,4]. Indeed, regular exercise training and physical activity reduce CVD risk in both primary and secondary prevention [5–8]. CR exercise reduces the morbidity and mortality from major CVD by ~20–25% [5]. In addition, it is associated with improvements in exercise capacity and all domains of physical performance after cardiac surgical intervention, which eventually results in a reduction in cardiac death endpoints [6–8]. Currently, CR exercise programs are used worldwide and have been incorporated into the infrastructure of hospitals.

Despite these reported benefits, the rate of outpatient participation in CR exercises remains disappointingly low, because of time constraints for hospital visits and the economic burden of participating [9–13]. Recently, research on the effectiveness of home-based or community based exercise programs has been performed by comparing them with hospital-based CR exercise; no difference in effectiveness was observed, especially in terms of the rate of recurrence of cardiac disease. During CR exercise, the intensity of exercise is important because the exercise has to be of an appropriate level. It has been pointed out that heavy exercise may actually increase the risk of CVD [5]. Exercise intensity is typically determined based on the measured heart rate (HR). For a given target heart rate zone (THZ), if the measured HR is greater than the THZ, then the exercise intensity is too high and should be reduced. However, if the measured HR is less than the THZ, then the exercise will be inefficient, and the patient needs to exercise more intensively. Thus, during CR exercise, measuring HR is an important factor in monitoring the patient’s exercise intensity. However, in home-based exercises, HR-measuring equipment, such as electrocardiography (ECG), is not readily available and effective exercises cannot be performed based on CR exercise guidelines [4,12,14,15]. Thus, there is a need for an HR-measurement-based CR exercise program that is simple and user-friendly to operate without requiring help from medical staff [15,16]. We have demonstrated a smartphone-based CR exercise program with no need for any external device [17,18]. It periodically measured HR by asking patients to place their finger on the built-in camera and then recommended the exercise intensity. However, the measured pulsatile signal during exercise can be corrupted by motion artifacts because of changes in the pressure or location of the fingertip on the camera lens. To make matters worse, the patient should hold the smartphone and repeatedly place a finger on the camera lens throughout the entire exercise session.

In this study, to address these issues, we developed a simple and user-friendly dedicated CR wearable sensor (DCRW) as a convenient watch-like device. To minimize motion artifacts, we used multiple photodetectors and singular value decomposition (SVD) to filter out uncorrelated signals corresponding to noise. Additionally, to effectively guide CR exercise, our DCRW automatically recommends the exercise intensity to the user by comparing the estimated heart rate (HR) with the target heart rate zone (THZ) in real time during exercise. The CR exercise includes pre-exercise, exercise with intensity guidance, and post-exercise periods. In the pre-exercise period, information such as THZ, exercise type, exercise stage order, and duration of each stage are set up using a smartphone application via Bluetooth communication. In the exercise period with intensity guidance, the DCRW continuously estimates HR using the reflected pulse signal from the wrist and compares the estimated HR with the THZ during exercise. Based on this comparison, the DCRW adjusts the exercise intensity to shift the patient’s HR to the THZ by indicating the HR status. In the post-exercise period, the DCRW transmits all the HR data to application via Bluetooth communication, and the user can monitor his/her own exercise history, including the ratio of the estimated HR to the THZ achieved.

## Methods

### Ethics statement

This study was approved by the institutional review board of Wonkwang University Hospital. All participants provided written informed consent (S1 and S2 Documents).

### Description of the DCRW-based system

#### Overview

**Fig 1:**
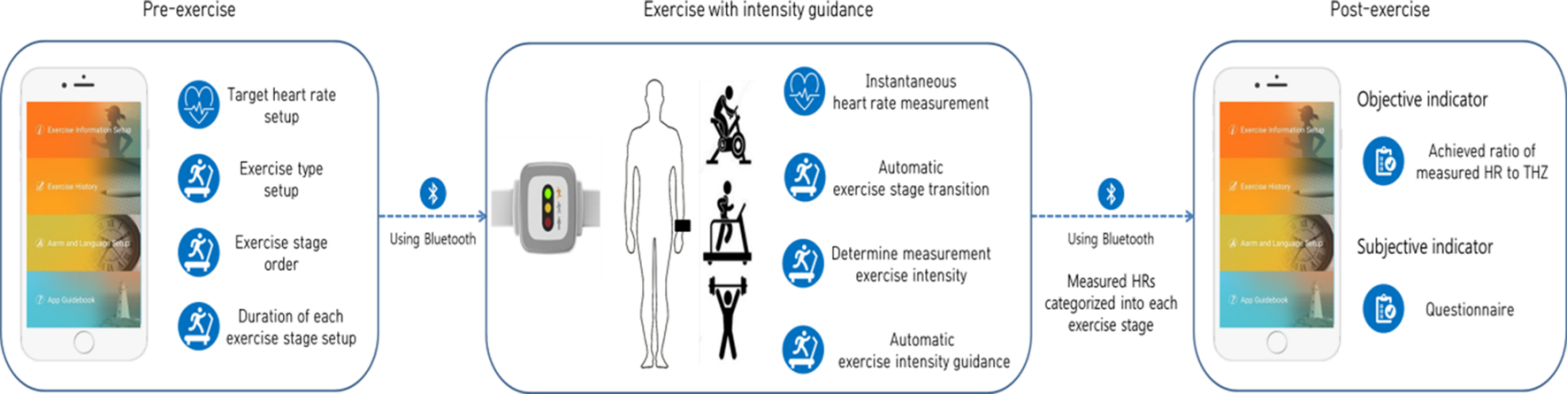
System for cardiac rehabilitation exercises using DCRW with a smartphone application.

Fig 1 illustrates the overall cardiac rehabilitation system using the DCRW and its several functions. CR exercise includes three steps: pre-exercise, exercise with intensity guidance, and post-exercise. In the pre-exercise step, exercise information, such as the THZ, exercise stage order, exercise type, and duration of each exercise stage, is set up using a smartphone application, which subsequently transmits this information to DCRW via Bluetooth communication. In the exercise with intensity guidance period, DCRW measures the heart rate in real time and provides feedback on exercise intensity to the patient in real time. In the post-exercise step, all measured HR data are sent to the smartphone application upon completion of the CR exercise.

#### Pre-exercise steps with the smartphone application

Before the CR exercise, a patient enters the THZ, exercise stage order, exercise type, and duration of each exercise stage using a smartphone application, which then transmits the information to DCRW via Bluetooth communication. The THZ has minimum and maximum allowed heart rate values during exercise. For successful CR exercises, it is important to determine the THZ, which can differ from patient to patient. Clinically, THZ can be determined with an exercise tolerance test (ETT) or a maximal exercise test that considers metabolism (METs), HR, blood pressure, respiratory exchange ratio (RER), and rating of perceived exertion (RPE), and determines the exercise intensity, including maximum heart rate *HR_max_*. Then, the THZ can be determined by multiplying the resulting *HR_max_* and the target intensity range (%), as shown in Table 1. The target intensity range is associated with the intensity of the exercise that a patient intends to perform the RPE, as recommended in the American College of Sports Medicine (ACSM) guidelines [4,19,20]. Alternatively, *HR_max_* can be found from various clinical investigations. Reference [21] recommends computing *HR_max_* as 207 - (0.7 × age) for a healthy person who has been performing exercise regularly, and 220 - age for a person with a low physical fitness level or requiring cardiac rehabilitation [4,21,22]. Reference [23] recommends 216.6 - (0.84 × age) for a person between 4 and 34 years old. Reference [24] recommends 208 - (0.7 × age) for a healthy person. Reference [25] recommends 206 - (0.88 × age) for a healthy female person above 50 to 60 years old. There are also other ways to calculate *HR_max_* [22]. Those are all guidelines that a patient can estimate his/her own *HR_max_*. However, for medical equipment purpose, *HR_max_* should be prescribed by a healthcare provider since it is subject-specific parameter. Clinically, *HR_max_* is generally obtained through a maximal exercise testing, and the exercise prescription is given to each patient based on the testing result. In this study, we used maximal exercise testing with the Q-Tel RMS program (Mortara Inc., Milwaukee, WI, USA), which is a telemetry monitoring system handling exercise parameters for CR monitoring [4,26,27]. All twenty participants first underwent maximal exercise testing, and the THZ was subsequently set between 50% and 70% of the prescribed *HR_max_* for the CR exercise.

**Table 1:** Intensity, HR_max_, and RPE [4,20].

Exercise Intensity	RPE	Target intensity (%)
Very, Very Light	6–8	< = 56
Very Light	9–10	57–60
Light	11–12	61–64
Moderate	13–14	70–76
Hard	15–16	81–86
Very Hard	17–18	91–96
Maximal	19–20	> = 97

Once the THZ has been determined, the patient chooses the exercise type, exercise stage order, and duration of each stage. The exercise stage order consists of warm-up, main exercise, rest, and cool-down, as recommended in the ACSM guidelines [4,19,20]. The main exercise stage can be split into multiple shorter stages: warm-up, main exercise, rest, further main exercise, and cool-down. For the warm-up and cool-down, walking or light stretching is recommended. The main exercise type can be outdoor cycling, indoor cycling, using of a treadmill, jogging, strength training, stair climbing, or rowing [4].

**Fig 2:**
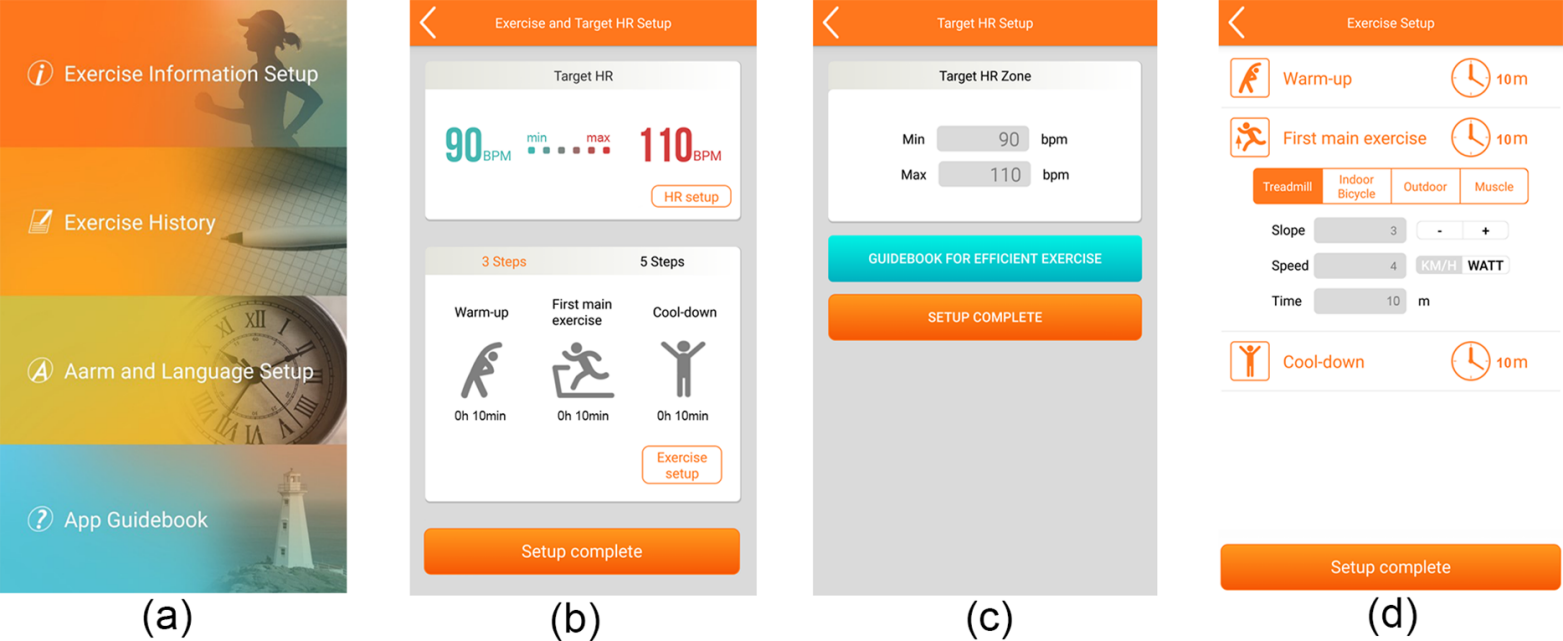
Pre-exercise stage with the cardiac rehabilitation (CR) application. (a) main menu, (b) exercise and THZ set-up, (c) THZ set-up (d) exercise type, exercise stage order, and duration of each stage.

The smartphone application we developed is available for iPhone and Android devices. Fig 2(A) shows the CR exercise main menu, which includes an “Exercise Information Set-up” button linked to the exercise set-up in Fig 2(B). By clicking the button “HR set-up”, the THZ can be set, as shown in Fig 2(C). Additionally, by clicking the button “Exercise set-up”, exercise type, exercise stage order, and duration of each stage can be set, as shown in Fig 2(D). Once all exercise information is set up, it is transmitted to the DCRW sensor via Bluetooth communication.

#### Exercise with intensity guidance stage

In the exercise with intensity guidance stage, the DCRW sensor measures HR continuously using the reflected pulse signal in a wrist from green LEDs and a photodiode, and compares the estimated HR with the THZ during exercise. Based on this comparison, the sensor adjusts the exercise intensity to shift the patient’s HR to the THZ by providing the patient with appropriate exercise intensity feedback during the exercise. The Beer-Lambert law states that the absorption of light as it passes through a sample is proportional to the thickness and the concentration of the sample, as follows:

−dI∝I∙Cdx, (1)

where *dI* is the infinitesimal change in light intensity as it passes through a sample of concentration *C* and thickness *dx*. Then, for a large sample,

$$I=I_{o}e^{-\alpha ∙C∙x}$$ (2)

where *I*_*o*_ is the intensity of the incident light, *α* is the absorption coefficient, and *x* is the thickness of the sample. The thickness of the wrist artery fluctuates as the heart beats. Correspondingly, the intensity of reflected light also fluctuates with the HR. The relative volumetric change in wrist artery changes the light absorption and, thus, can be used to produce a photoplethysmogram (PPG).

**Fig 3:**
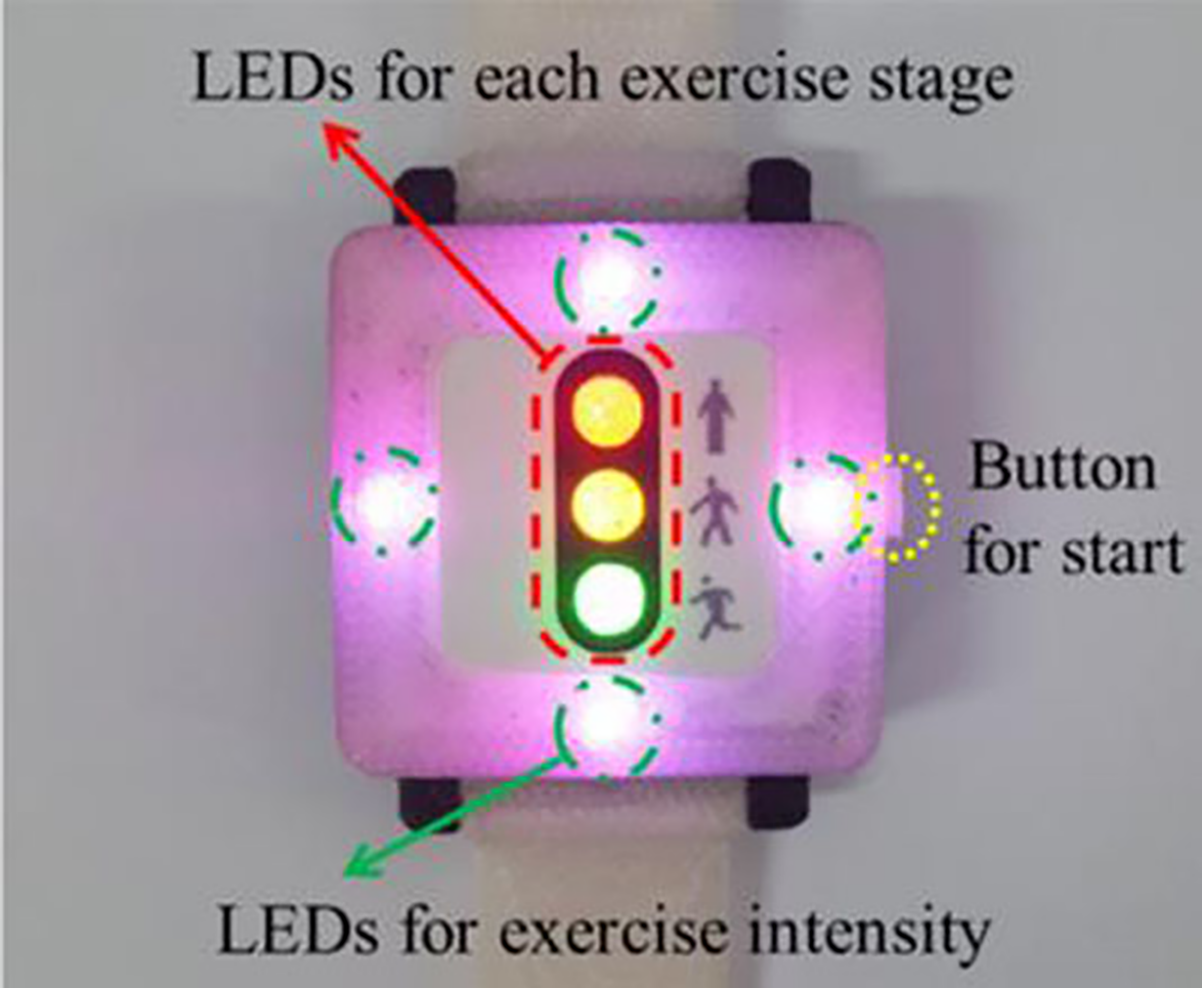
The DCRW front face.

**Fig 4:**
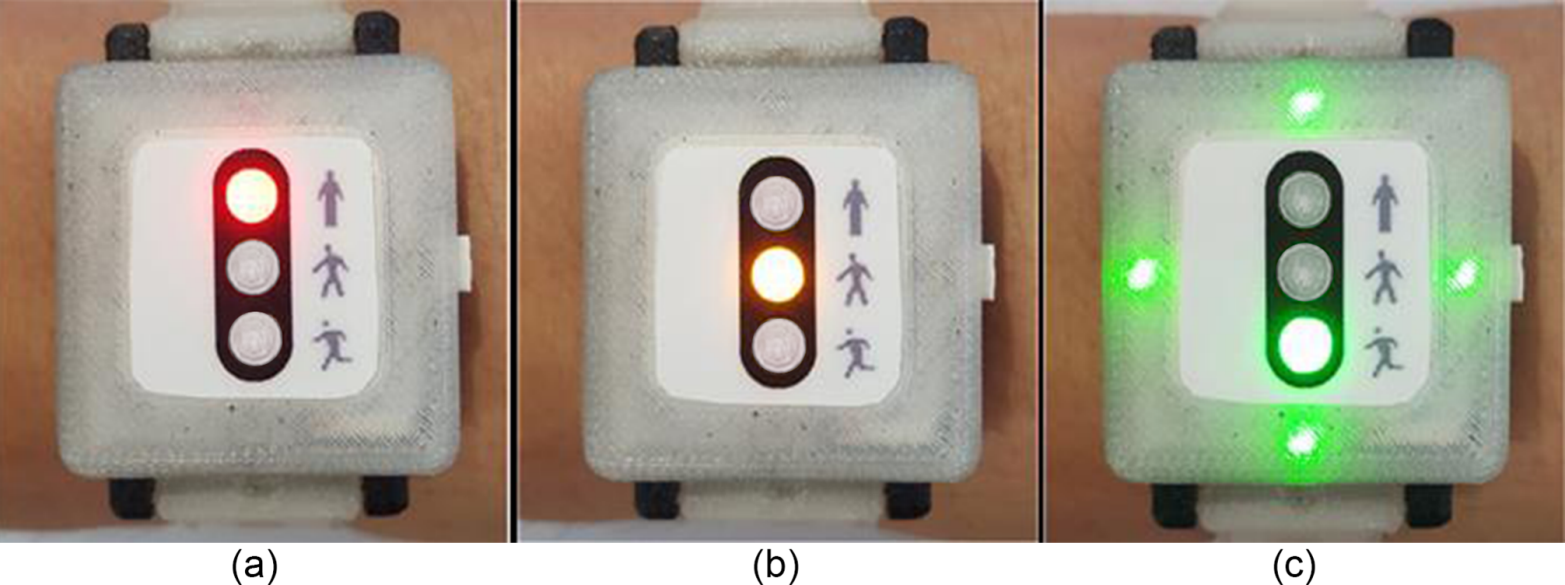
Middle LED part providing information on exercise stage. (a) before starting exercise or during rest (b) warm-up or cool-down stage (c) main exercise stage.

In the DCRW we developed, two sets of LEDs (middle and side parts) are deployed on the face front (Fig 3). The middle LEDs (traffic light concept) consist of three LEDs of red (top), yellow (middle) and green (bottom), which provide information on the exercise stage status: red LED for before exercise or during rest, yellow LED for warm-up or cool-down, and green LED for main exercise. In the beginning, the red LED is turned on as the DCRW device is turned on by clicking the start button on the right side (Fig 4(A)). When the patient clicks the button again, the CR exercise starts. Then, the red LED turns off and the yellow LED turns on (Fig 4(B)), corresponding to the warm-up stage. When the warm-up stage is finished, the main exercise starts immediately and the green LED turns on automatically while the yellow LED turns off (Fig 4(C)). Additionally, whenever the exercise stage changes, the DCRW vibrates for two seconds. After the main exercise stage is finished, the green LED turns off and another LED turns on depending on the next stage: red for rest and yellow for cool-down. In this way, the middle LED part provides the patient with exercise stage information.

**Fig 5:**
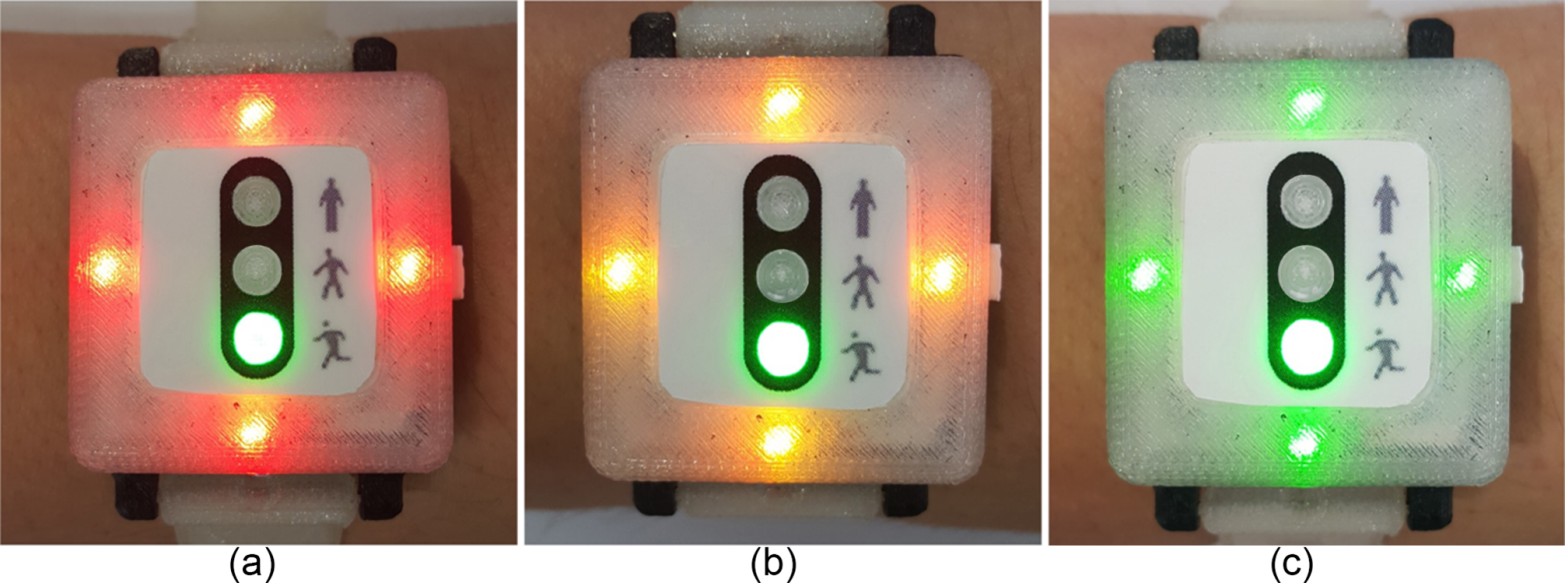
Side LEDs for exercise intensity guidance. (a) pace down (HR is greater than THZ), (b) pace up (HR is less than THZ), (c) keep the pace (HR is within THZ).

During the main exercise period, the DCRW measures the heart rate in real time and compares the measured HR with the THZ. If the measured HR is greater or less than the THZ, the DCRW indicates an alarm to the patient via other LEDs, at the top, bottom, and left and right sides (Fig 3). On each side, red, yellow, and green LEDs are used. If the measured HR is greater than the THZ, the red LEDs on the four sides blink (Fig 5(A)), informing the patient to reduce the pace, to decrease the HR. If the HR is less than THZ, the yellow LEDs on four sides blink (Fig 5(B)), informing the patient to step up the pace to increase the HR. Otherwise, the green LEDs on four sides blink (Fig 5(C)), telling the patient to keep the pace. These alarm signals help the patient to adjust the exercise intensity to move the patient’s HR into the THZ by providing appropriate exercise intensity feedback during the exercise. Note that the DCRW also vibrates for two seconds when the exercise intensity is recommended. This vibration functionality aims to prevent the exercise interference.

#### Post-exercise stage

**Fig 6:**
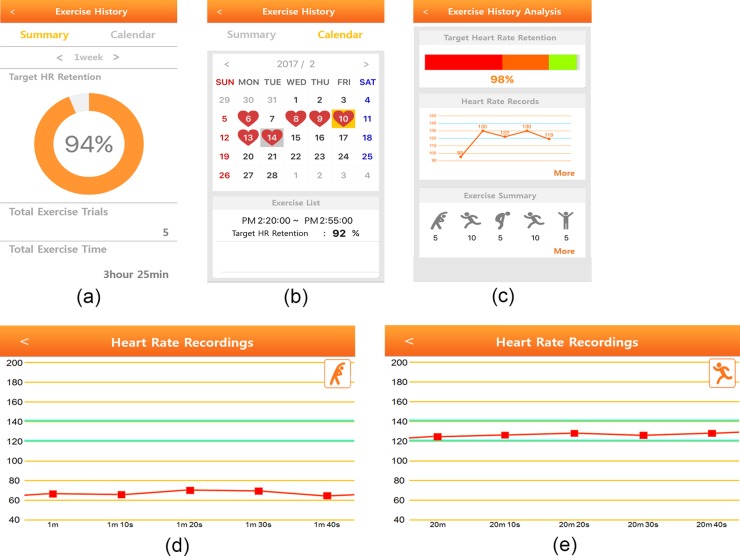
Post-exercise stage with the cardiac rehabilitation (CR) application. (a) exercise history summary, (b) calendar-based exercise history, (c) exercise analysis, (d) heart rate trace example in warm-up stage, (e) heart rate example in main exercise stage.

In the post-exercise stage, all the measured HR data are sent to the smartphone application upon completion of the CR exercise. The HRs are categorized into the exercise stages of warm-up, main exercise, rest, and cool-down, as set up in the pre-exercise stage. Fig 6(A) shows the exercise summary during a certain period (e.g., 1 week, 1 month). It includes the total number of exercise trials and times. Additionally, the ratio of measured HR to THZ achieved is displayed as an objective indicator for evaluating the exercise. Fig 6(B) shows a calendar-based exercise summary, where the red heart is marked on the exercise trial day. On clicking the exercise trial day, more detailed exercise information is provided on exercise stage order, exercise type, duration of each exercise stage, and the ratio of measured HR to THZ achieved (Fig 6(C)). Furthermore, the application provides HR traces along with THZ on clicking the ‘more’ button. Fig 6(D) and 6(E) are examples of HR traces along with THZ in the warm-up stage and main exercise stages, respectively.

Furthermore, the user completes a questionnaire using scales for chest pain, dyspnea, and leg pain during the exercise, which can be used as subjective indicators for evaluating the exercise. Thus, in the post-exercise step, the pre-exercise set-up information (e.g., THZ, exercise type, exercise stage order, and duration of each stage) and all measured HR data, including the ratio of measured HR to THZ, achieved during the main exercise can be monitored by the patient and, potentially, by clinicians too.

### Motion artifact reduction in DCRW

In the DCRW-based CR system, the most important issue is to accurately measure HR during exercise. To increase the accuracy of HR measurements, we assessed both hardware and software. From the perspective of hardware, we used a multichannel sensor consisting of multiple green LEDs and multiple photodetectors. Regarding the software, we used the multiple signals acquired in a truncated singular value decomposition method (SVD) to extract a clean signal, leading to an accurate measured HR.

### Multichannel sensor

**Fig 7:**
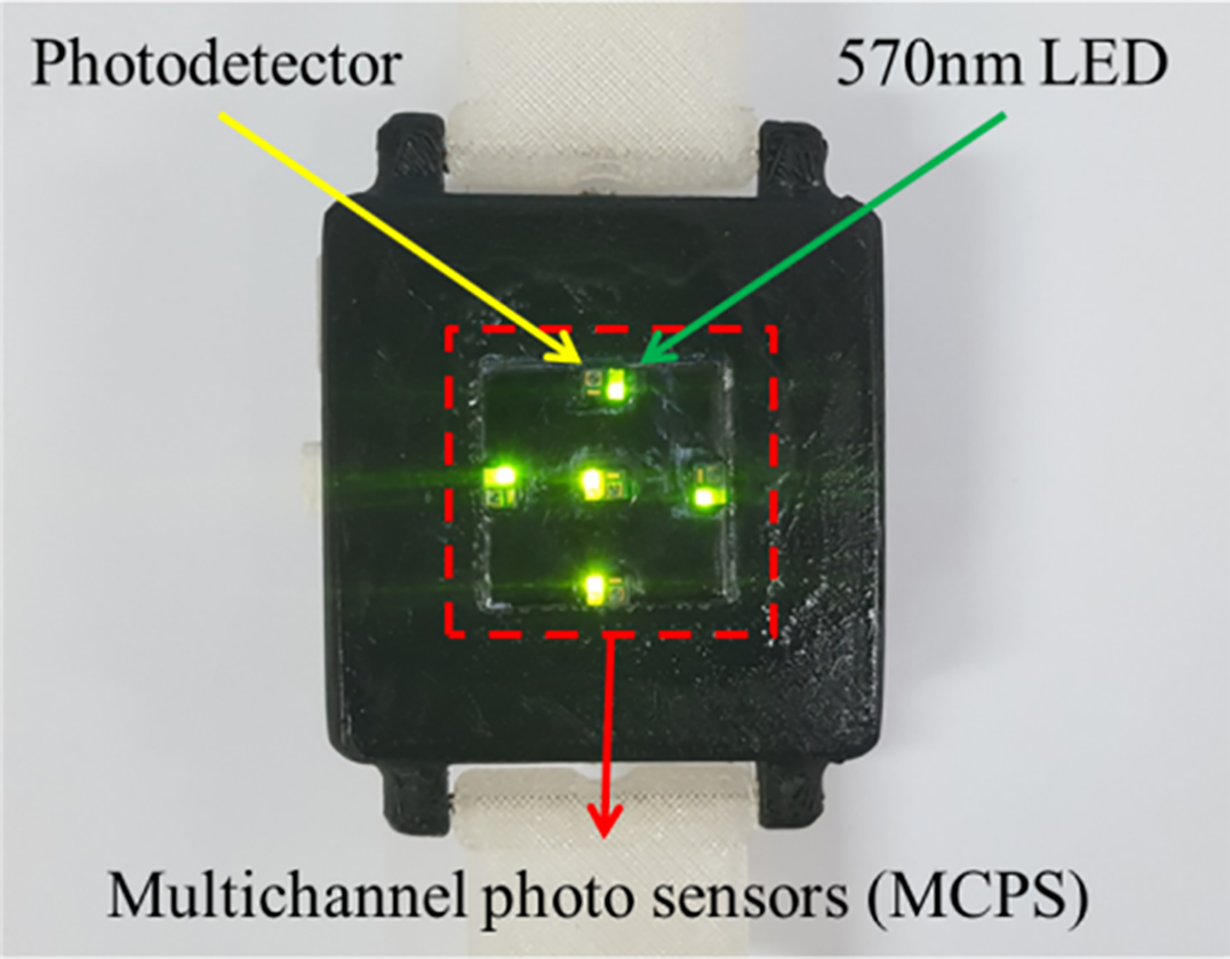
DCRW bottom view.

In the DCRW, we acquired multiple PPG signals simultaneously using multichannel photosensors (MCPS). We used the NJL5303R photosensor, which includes a photodetector and 570 nm (green) LED. Five photosensors were used (Fig 7). The distance between the individual sensors was 7 mm. Each photosensor was acrylic coated with a 1 mm protruding shape and the DCRW base (background) was painted black for optical and sweat shielding. The watch appearance was printed with polylactic acid (PLA) material by a 3D printer (3DP-110F, HyVISION SYSTEM Inc., Republic of Korea) via SolidWorks (SolidWorks 2013, SolidWorks Corp., USA).

**Fig 8:**
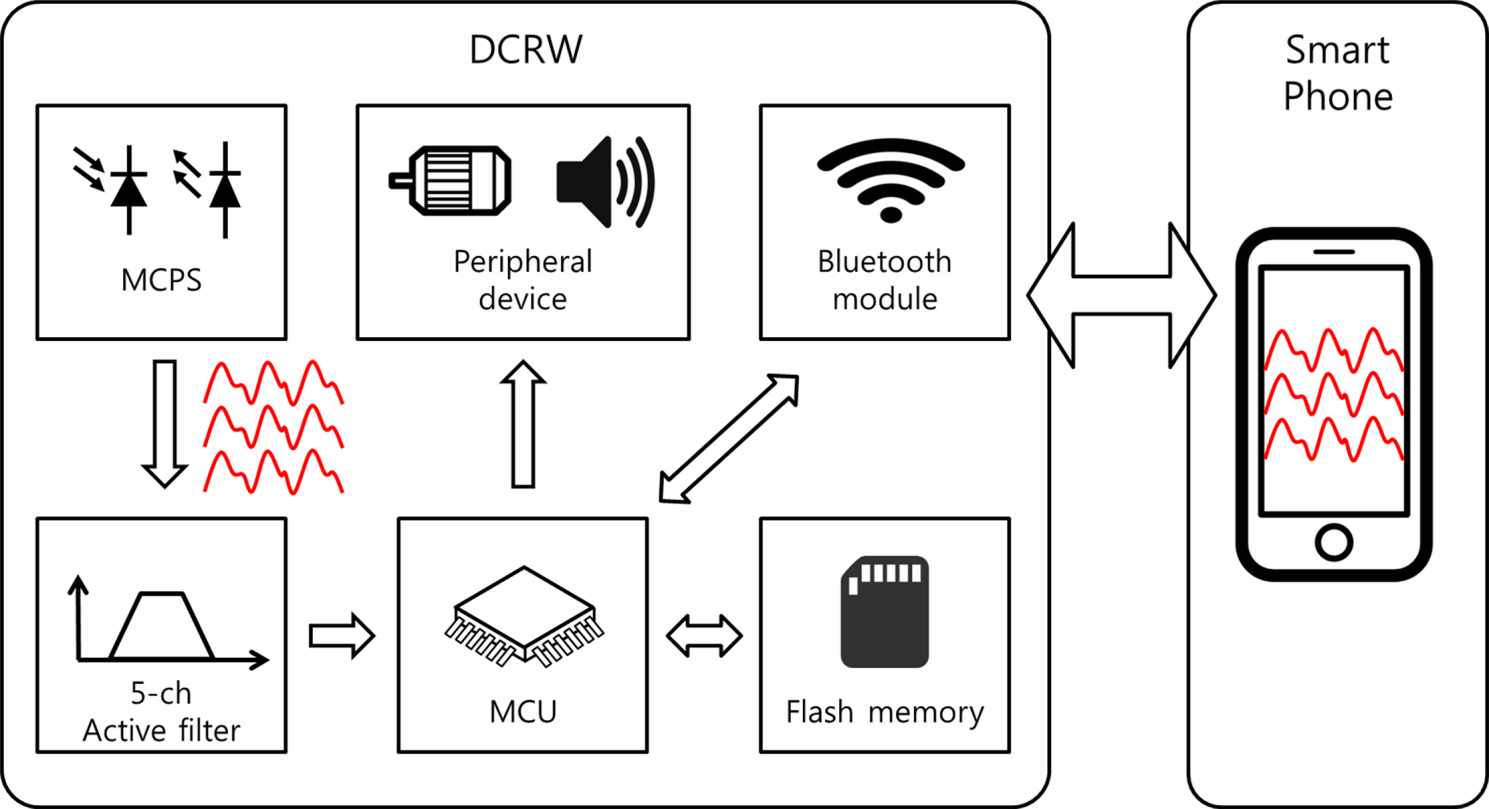
Internal system block diagram of the DCRW.

Fig 8 shows the internal system block diagram. The LED driving circuit for the current supply included a metal oxide silicon field-effect transistor (MOSFET) and a digital-to-analog converter (DAC) to control the current. The brightness of the LED changes according to the value of the DAC, and the reflected PPG signal amplitude can be adjusted according to the brightness. Each PPG signal obtained from the photosensor is converted to a voltage signal through trans-impedance amplifiers. Subsequently, the converted small voltage signal with noise passes through amplification and filtering via an analog filter. Each voltage signal was amplified and filtered using an active filter (MCP6004, Microchip) with a cut-off frequency of 0.5–10 Hz, which prevents from signal saturation and distortion. The response type of the designed filter was 4^th^-order infinite impulse response (IIR) Butterworth, which is with 2^nd^-order low pass filter (LPF) and 2^nd^-order high pass filter (HPF). The filtered signal was converted to digital data using a 12-bit analog-to-digital converter (ADC) built into the microcontroller unit (TM4C123GH6PMI, Texas Instruments). The digital data were converted at a 100 Hz sampling rate. Digital data were stored in a 64M-bit flash memory (S25FL164K, Spansion) and can be communicated via Bluetooth (HM-11, JNHuaMao Technology). The power required for the MCPS was designed with a low dropout regulator with 3.3-V output.

### Pulse signal reconstruction with singular value decomposition

Given the five acquired multiple pulse signals, we can denote each channel pulse signal by p_k_(n), where *k* = 1, 2, 3, 4, and 5 for each channel, 1 to 5. We arranged each signal p_k_(n) as a two-dimensional matrix ***P***, which can be expressed as

$$P=\left[\begin{array}{ccccc} p_{1}\left(1\right) & p_{2}\left(1\right) & p_{3}\left(1\right) & p_{4}\left(1\right) & p_{5}\left(1\right)\\ p_{1}\left(2\right) & p_{2}\left(2\right) & p_{3}\left(2\right) & p_{4}\left(2\right) & p_{5}\left(2\right)\\ \vdots & \vdots & \vdots & \vdots & \vdots \\ p_{1}\left(N\right) & p_{2}\left(N\right) & p_{3}\left(N\right) & p_{4}\left(N\right) & p_{5}\left(N\right) \end{array}\right]$$ (3)

where each row corresponds to each channel pulse signal. Then, we applied singular value decomposition (SVD) as follows,

$$P=U\Sigma \;V^{T}$$ (4)

where ***U*** and ***V*** are the left and right singular vectors, respectively, and ***Σ*** corresponds to singular values of matrix ***P***. More specifically, the eigenvectors of ***PP***^***T***^ make up the columns of ***U*** (*N* × *N* matrix), and the eigenvectors of ***P***^***T***^ ***P*** make up the columns of ***V*** (5 × *5* matrix). The singular values *σ*_*i*_ in ***Σ* (***N* × *5* diagonal matrix) are square roots of the eigenvalues from ***PP***^***T***^ or ***P***^***T***^ ***P*** as

$$\Sigma =\left[\begin{array}{ccccc} \sigma_{1} & 0 & 0 & 0 & 0\\ 0 & \sigma_{2} & 0 & 0 & 0\\ 0 & 0 & \sigma_{3} & 0 & 0\\ 0 & 0 & 0 & \sigma_{4} & 0\\ 0 & 0 & 0 & 0 & \sigma_{5}\\ \vdots & \vdots & \vdots & \vdots & \vdots \\ 0 & 0 & 0 & 0 & 0 \end{array}\right]$$ (5)

**Fig 9:**
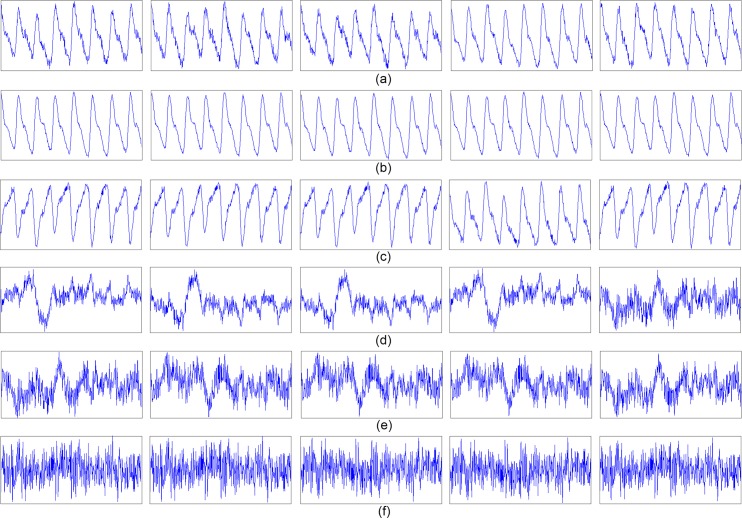
Multichannel pulse signal measured from DCRW and its singular decomposition value-based decomposed signals (channels 1 to 5 from left to right). (a) measured multichannel pulse signals, (b) decomposed signals *u*_1_*σ*_1_*v*_1_^T^, (c) decomposed signals *u*_2_*σ*_2_*v*_2_^T^, (d) decomposed signals *u*_3_*σ*_3_*v*_3_^T^, (e) decomposed signals *u*_4_*σ*_4_*v*_4_^T^, (f) decomposed signals *u*_5_*σ*_5_*v*_5_^T^.

To illustrate how to de-noise the signals with the multiple channels using SVD, we used our DCRW on a subject’s wrist for pulse signal acquisition. Fig 9(A) shows the five multiple pulse signals measured from each channel for 5 s, where the signals from left from right are from channels 1–5, respectively. Using SVD, the resulting singular values *σ*_1_, *σ*_2_, *σ*_3_, *σ*_4_ and *σ*_5_ were obtained as 1.25×10^5^, 2.03×10^3^, 528, 320, and 222, respectively. Then, the information energy of the first singular value ($\sigma_{1}^{2}$) was 99.98% of the information energy from the total singular values ($\sum_{i=1}^{5}\sigma_{i}^{2}$). Fig 9(B)–9(F) show the all decomposed signals: u_1_σ_1_v_1_^T^ in Fig 9(B), ***u***_2_*σ*_2_***v***_2_^***T***^ in Fig 9(C), ***u***_3_*σ*_3_***v***_3_^***T***^ in Fig 9(D), ***u***_4_*σ*_4_***v***_4_^***T***^ in Fig 9(E), and ***u***_5_*σ*_5_***v***_5_^***T***^ in Fig 9(F). The results show that the dominant singular value *σ*_1_ with respect to the ***v***_**1**_ and ***u***_**1**_ forms the principal component in all the channel pulse signals. Thus, de-noising can be performed with the truncated SVD as

$$\hat{P}=U_{tr}\Sigma_{tr}{V_{tr}}^{T},$$ (6)

where the principal component ***V***_***tr***_ = {***v***_1_}, with associated scaling vectors ***U***_***tr***_***Σ***_***tr***_ = {***u***_1_*σ*_1_}. Additionally, the sizes of ***U***_***tr***_, ***Σ***_***tr***_ and ***V***_***tr***_ are reduced to *N* × 1, 1 × 1 and 5 × 1, respectively. This means that the truncated SVD performs not only signal de-noising but also data compression. In terms of data compression, the data size of P is 5*N* while the data size of the truncated SVD (***U***_***tr***_, ***Σ***_***tr***_ and ***V***_***tr***_) is *N*+6. In the example of Fig 9, *N* was 500; thus, the data size was reduced from 2,500 to 506.

Let us denote the reconstructed pulse signals by ${\hat{p}}_{k}(n)$, where *k* denotes the channel index, from 1 to 5. With ${\hat{p}}_{k}(n)$, we calculated the percent root mean square difference (PRD), which evaluates the difference between each measured pulse signal p_k_(n) and each reconstructed pulse signal ${\hat{p}}_{k}(n)$ as

$${PRD}_{k}=\sqrt{\frac{\sum_{j=1}^{N}{\left(p_{k}(n)-{\hat{p}}_{k}(n)\right)}^{2}}{\sum_{n=1}^{N}{\left(p_{k}(n)\right)}^{2}}}\times 100.$$ (7)

Subsequently, we chose the ‘best’ channel, providing the lowest PRD, and used the corresponding reconstructed pulse signal for the HR calculation, the algorithm for which incorporates a filter bank with variable cut-off frequencies, spectral estimates of the HR, rank-order non-linear filters, and decision logic [28]. The HR calculation was done with each 5-s segment with 50% overlap.

To evaluate the HR estimation based on our DCRW with truncated SVD, we used a modified version of the Bruce protocol, which consists of 5 min of walking for a warm-up, 10 min of jogging, 5 min of rest (walking), an additional 10 min of jogging, and 5 min of walking for cooling down, all on a treadmill. For the first session of jogging, the slope was 12° and the speed was 4.0 km/h. For the second session of jogging, the slope and speed were increased slightly, to 13° and 5.4 km/h. For HR estimation, twenty subjects who presented for cardiac rehabilitation exercises at Wonkwang University Hospital were recruited by trained study personnel. In total, 11 men and 9 women with an average age of 32.1±6.3 years participated. During the exercise, one trained study personnel (Se-Eung Noh) and two engineers (Hoon Ko and Heewon Chung) monitored the real-time pulse signal from a smartphone via Bluetooth communication. We also monitored each subject’s movement and status and wrote the memo when any one of channel is corrupted by motion artifacts. Furthermore, we recorded the all raw data in the flash memory, and confirmed that our proposed algorithm is effective under motion artifacts. Our protocol for data collection and analysis was approved by the institutional review board of Wonkwang University Hospital.

### Data availability

All relevant data have been uploaded to Figshare: https://dx.doi.org/10.6084/m9.figshare.5001920.v1.

## Results

### Truncated SVD based HR estimation

To evaluate the estimated HR values, ECG data were recorded simultaneously sing a 24-h Holter monitor (SEER Light, GE Healthcare, Milwaukee, WI, USA). For the error analysis, we used mean absolute error (MAE) and root mean squared error (RMSE) for each subject, defined as follows:

$$RMSE=\sqrt{\sum \frac{{\left(Y_{DCRW(i)}-Y_{holter(i)}\right)}^{2}}{N}}$$ (8)

where *Y*_*DCRW*_ is the HR (bpm) estimated from the DCRW at the *i*^*th*^ segment, and *Y*_*holter*_ is the HR (bpm) from the Holter at the *i*^*th*^ segment. We compared the data with two further approaches: single channel-based HR measurements and a multiple channel-based template update method [29], which found the best quality single channel based on a template update and correlation method. For statistical difference, one-way analysis of variance (ANOVA) with Bonferroni multiple comparison test (*p*<0.05) was used using SPSS ver.18 (SPSS Inc., Chicago, IL, USA).

**Fig 10:**
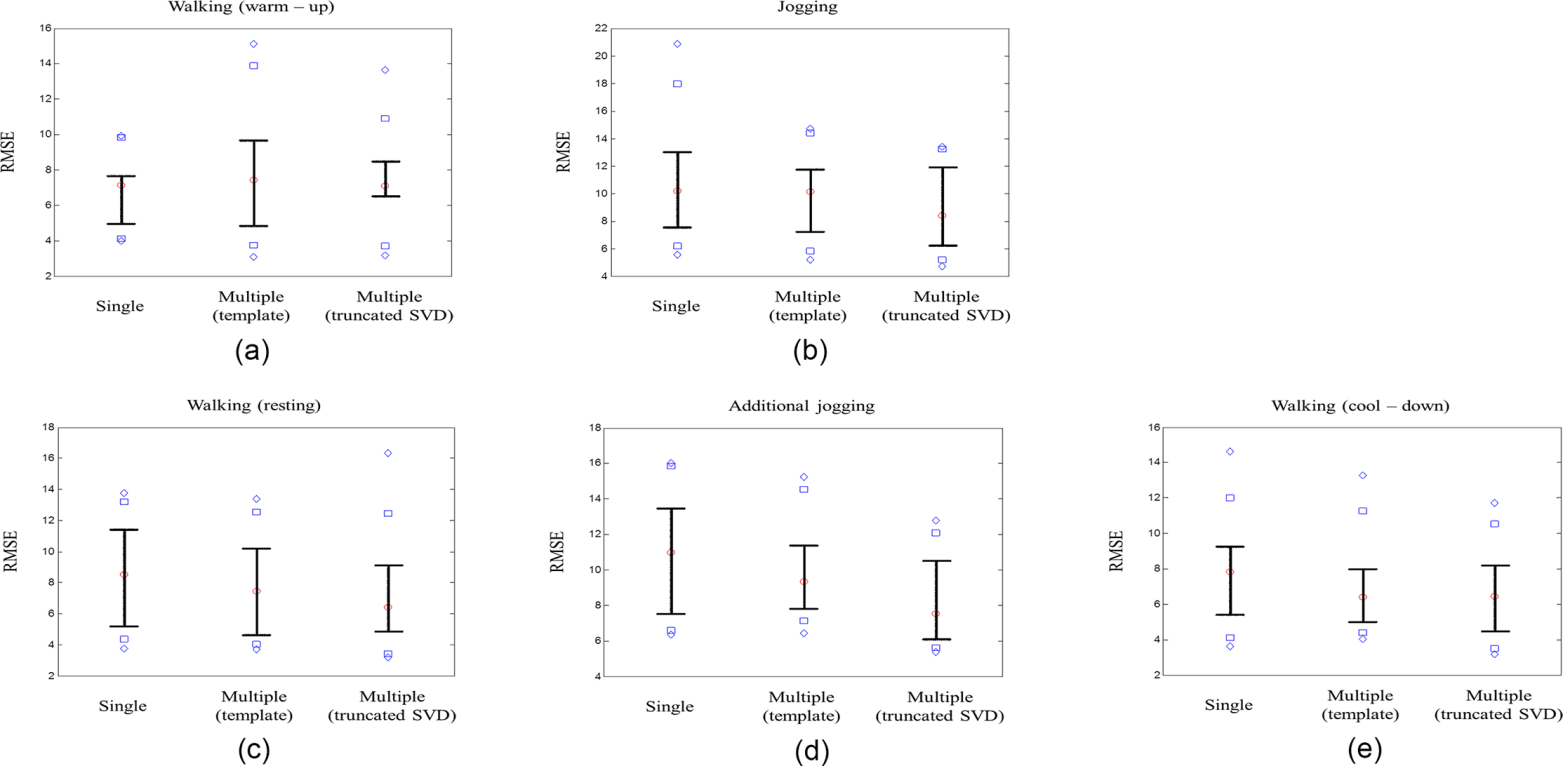
RMSEs distribution of the single-channel method, multiple channel-based template update method, and the proposed method with our DCRW. (a) walking (warm-up), (b) jogging (main exercise), (c) walking (rest), (d) additional jogging (main exercise) and (e) walking (cool-down). The diamonds above and below represent the 5^th^ and 95^th^ percentiles of each group, and the squares above and below represent the 90^th^ and 10^th^ percentiles. Whiskers above and below indicate the 75^th^ and 25^th^ percentiles, respectively. The circle is the median value.

Fig 10 shows the RMSEs distribution of the single-channel method, multiple channel-based template update method, and the proposed method with our DCRW for walking (warm-up), jogging (main exercise), walking (rest), additional jogging (main exercise), and walking (cool-down). The diamonds above and below represent the 5^th^ and 95^th^ percentiles for each method, and the squares above and below represent the 90^th^ and 10^th^ percentiles. Whiskers above and below indicate the 75^th^ and 25^th^ percentiles, respectively. The circle is the median value. Table 2 summarizes the mean and standard deviation of RMSEs and statistical significance for the entire exercise stage. For the median and standard deviation of RMSEs in Table 2, our proposed method with the DCRW was lower than the two other methods for all exercise stages. Especially in the two main exercise stages, the results from DCRW were significantly different from the other two methods (*p* < 0.05). More specifically, during the first main exercise stage, the median of the RMSEs of our proposed method with DCRW was 1.22 and 1.21 times smaller than those of the single-channel method and the multiple channel-based template update method, respectively. Additionally, the standard deviation of the RMSEs of our proposed method with DCRW was 1.49 and 1.01 times smaller than those of the single-channel method and the multiple channel-based template update method, respectively. Similarly, during the second main exercise stage, the median of the RMSEs of our proposed method with DCRW was 1.46 and 1.24 times smaller than those of the single-channel method and the multiple channel-based template update method, respectively. The standard deviation of the RMSEs of our proposed method with DCRW was 1.26 and 1.10 times smaller than those of the other two methods, respectively.

**Table 2:** Performance comparison of the single channel, multiple channel with template updates, and multiple channel with truncated SVD. Median and standard deviation of root mean square errors (RMSEs) for walking (warm-up), jogging (main exercise), walking (warm-up), additional jogging (main exercise) and walking (cool-down).

Stage Method	Walking (warm-up)		Jogging		Walking (resting)		Additional jogging		Walking (Cool-down)	
	Median of RMSE	Standard deviation of RMSE	Median of RMSE	Standard deviation of RMSE	Median of RMSE	Standard deviation of RMSE	Median of RMSE	Standard deviation of RMSE	Median of RMSE	Standard deviation of RMSE
Single	7.1249	1.9555	10.1756	4.5278	8.5354	3.3805	10.9654	3.4941	7.8312	3.0539
Multiple (template)	7.4302	3.6887	10.1196	3.0596	7.4682	3.2133	9.3584	2.7648	6.4168	2.6421
Multiple (truncated SVD)	7.0868	2.8104	8.3765	3.0328	6.4025	3.8279	7.5337	2.5108	6.4253	2.6401

### Exercise intensity alarms

**Fig 11:**
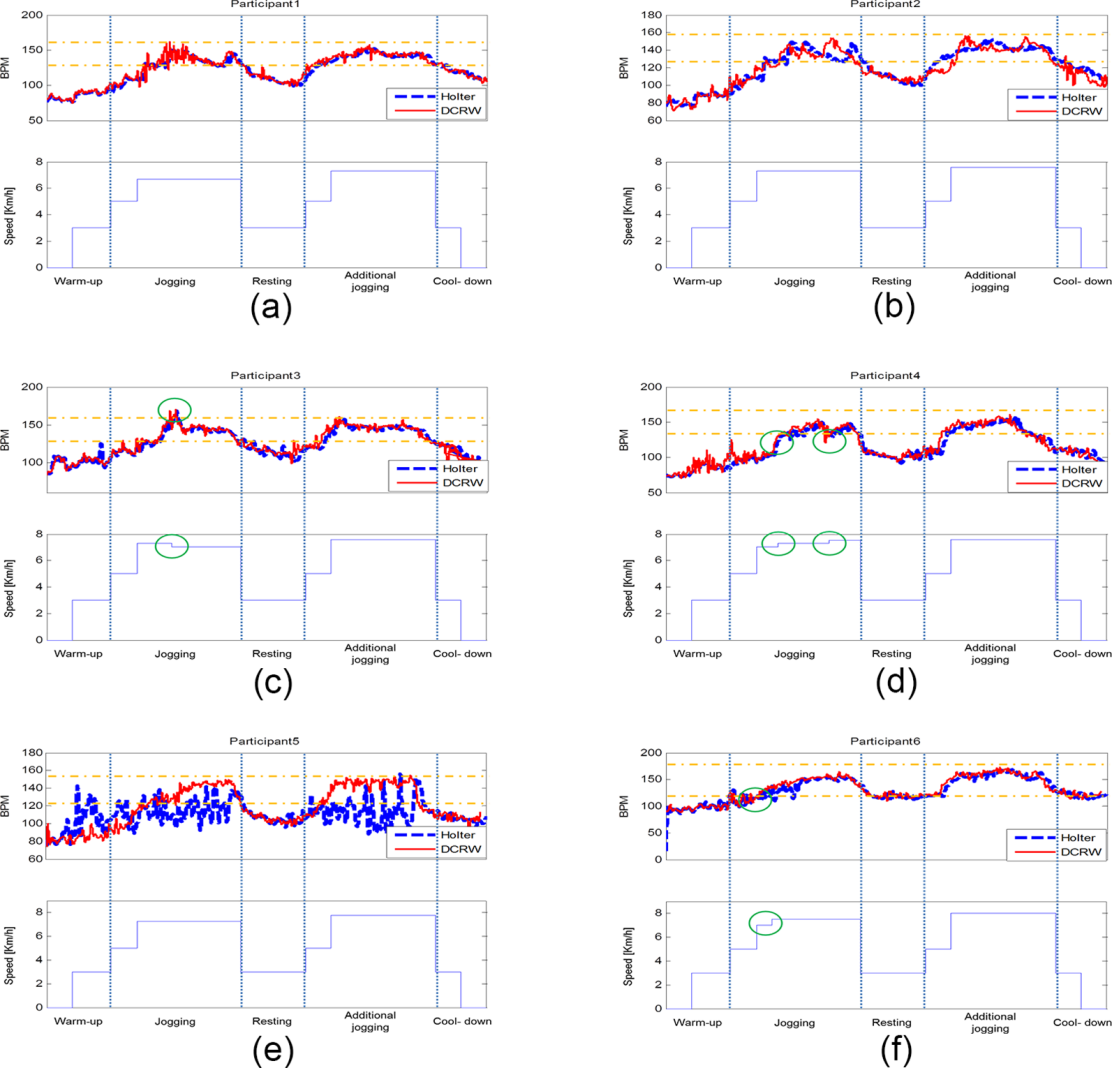
Individual HR traces obtained with the Holter monitor and DCRW during exercise. (a) subject #1, (b) subject #2, (c) subject #3, (d) subject #4, (e) subject #5, (f) subject #6.

During the main exercise stage, the DCRW compares the measured HR with the THZ, and informs the subject about the appropriate exercise intensity by blinking different color LEDs on the side. Fig 11 shows HR traces obtained from the Holter monitor and our method with the DCRW for six subjects during the exercise stages. The complete results with additional 14 subjects are described in the supplementary materials. In the example shown in Fig 11(A), at the beginning of the main exercise, the measured HR was lower than the minimum of THZ, causing the yellow LEDs to blink, informing the patient to increase the pace. The patient speeded up and the HR was within the THZ. Then, the blinking yellow LEDs switched to green LEDs. In the middle of the main exercise, HR was greater than the THZ maximum, causing the red LEDs to blink, informing the patient to slow down. The patient did so, slightly, and the HR was again within the THZ. Thus, the blinking red LEDs switched to green LEDs. During the additional main exercise, DCRW observed one moment when the HR was greater than the THZ maximum, and informed the patient to slow down by blinking the red LEDs again. All twenty patients successfully completed CR exercises adjusting HR within the THZ.

## Discussion

We developed a DCRW that automatically informs the user of an appropriate level of exercise for keeping the HR within the THZ. Additionally, the DCRW informs the user about exercise stage state and provides the capability for measuring HR during exercise. A key issue is to accurately estimate HR even with motion artifacts during exercise. To achieve accurate HR measurements, from a hardware perspective, we used multichannel photosensors rather than a single-channel photosensor, as is normally used in devices from Apple, Fitbit, and Samsung. By acquiring multiple pulse signals from multiple positions, we improved the chance of acquiring a clean signal. From this, the next issue concerns how the multiple signals should be handled. One possible solution is to select the ‘best’ signal or channel among them [29]. The solution can work effectively if at least one channel is clean, with fewer motion artifacts. Furthermore, this selection should be correct. Another possible solution is to weight each channel signal and combine them into a single signal. This solution can also work, assuming the weighting constants are accurate. However, these two approaches require a clean signal template, which can be obtained from motionless conditions or generated artificially. Subsequently, the measured multiple channel signals are evaluated with the template to select the best signal or to weight all the channel signals. Furthermore, if the template is not correctly formed, then the results will not be provided as expected. Indeed, during exercise, the pulse shape in amplitude and pulse width changes dynamically. Thus, the time-varying pulse shape can be an obstacle for a channel selection or weighting approach. Our method was to apply truncated SVD for signal de-noising. The signal decomposition capability of SVD was exploited to extract the significant feature components of the pulse signals by decomposing the signal into a set of basic patterns with associated scaling factors. We showed that the pure pulse was concentrated mostly within the first singular value *σ*_1_, with related singular vectors ***u***_1_ and ***v***_1_. Consequently, only the relevant parts of the singular triplets need to be remained, which also provide a benefit in compressing the data.

However, the truncated SVD method cannot correctly de-noise the signals when all channels are severely corrupted by motion artifacts with a very low SNR, especially during high-intensity exercise, which causes marked differences in pressure and displacement between the photosensor and the measurement sites for all channels. If overwhelming motion artifacts are present in all channels, the truncated SVD will result in the same pattern of overwhelming motion artifacts as the denoising result. On the other hand, if the overwhelming motion artifacts are different among the channels, all singular values become similar, which does not provide the significant feature component of the signals from SVD. Thus, we should carefully consider the contact issue. Especially for medical use of high-fidelity wearable devices, maintenance of a constant pressure and displacement between the photosensor and the measurement site is critical. To minimize the motion artifacts caused by inappropriate contact, we may tighten the sensor by simply using adhesive or/and wrapping tape. We believe that research effort should focus on the hardware or accessory issue, which resolves the contact issue. During the exercise tests on twenty subjects, we did not consider the contact issue since the inappropriate contact is also one of the motion artifact factors to be investigated. However, to be more complete form of DCRW, the contact between photosensors and a wrist should be automatically confirmed. Then, it is required to include additional step to confirm that the pulse is correctly measured before the de-noising step. In addition, to obtain better signals for more accurate HR, we may consider flexible materials based DCRW, which makes the contact more tightly on a wrist. To use the flexible materials for a wearable device, it is required to fabricate flexible and stretchable electronics. Recently, there have been much research efforts in flexible electronics: to introduce new flexible materials or make conventional devices flexible by substrate thinning. Nevertheless, the new materials are yet technologically immature with lower performance, and the substrate thinning techniques are complicated and expensive [30,31]. We believe that the techniques for flexible electronics will be mature with higher performance in the future.

ACSM recommends that CR exercise can be performed with warm-up, main exercise and cool-down for a total of 30 to 40 minutes and four to five times per week [4]. For the medical purpose, the exercise orders, types and duration need to be confirmed by healthcare providers. If a patient gets the exercise prescription and sets the parameters in DCRW, they are stored and can be default parameters for the next CR exercise. Clinically, every three to six months, a healthcare provider prescribes the CR exercise parameters such as *HR*_*max*_, THZ, exercise orders, types and duration. For future research, we may consider automatic recommendation system of the exercise parameters based on the track of exercise tendency because the parameters are subject-specific. We believe that our proposed DCRW and its application attract many researchers in the fields of cardiac rehabilitation and/or artificial intelligence, and pave the way for the automatic subject-specific exercise prescription based on exercise history such as exercise parameters, HR traces and the ratio of the HR to the THZ achieved.

The DCRW is available when a patient is still in hospital, after undergoing treatment for a heart attack or other cardiac condition. It is also available once a patient has left the hospital or at any other time to help prevent future heart problems. Furthermore, it is available for healthy individuals, where it can ensure efficient exercise by indicating an appropriate exercise intensity. For future research, we are recruiting more subjects, including patients with cardiac diseases and healthy subjects, to rigorously validate the DCRW we developed. We believe that the DCRW can be extended to remote diagnosis, which can provide information to the physician in charge of a patient’s CR exercise progress. Additionally, the DCRW can be modified further to address lifestyle changes, education, and emotional support for more effective cardiac rehabilitation.

## Conclusions

We described a wearable sensor, the DCRW, which we developed to provide effective CR exercises. To measure HR accurately, we developed multichannel photosensors and used a truncated SVD algorithm. We also used two sets of LEDs to inform the patient about exercise stage status and appropriate exercise intensity during exercise. Along with the DCRW, we developed a smartphone application, which can set and monitor exercise information and history in pre-exercise and post-exercise steps. As a pilot study, we showed clinical potential that the DCRW can be positively applied for CR exercise. In the future research, we plan to investigate how the DCRW is effective in lowering the recurrence of CVD. We will also consider improvements in exercise capacity and all domains of physical performance after cardiac surgical intervention. We believe that our proposed DCRW could gain wide acceptance as a home-based clinical CR exercise tool with the advantages of good accessibility, low cost, and ease of use after the clinical studies.

## Supporting information

S1 Document Original statements and written consents. The statements and written consents in Korean.
(DOCX)

S2 Document Translated statements and written consents. The translated statements and written consents in English.
(DOCX)
