# Supplementary material for: Dedicated cardiac rehabilitation wearable sensor and its clinical potential
Source: PLoS One. 2017 Oct 31;12(10):e0187108. doi: 10.1371/journal.pone.0187108 (PMC5663433; doi:10.1371/journal.pone.0187108)
Supplement: S1 Document — The statements and written consents in Korean. (DOCX) [file pone.0187108.s001.docx]

**대상자 설명문 및 서면 동의서**

1. 연구 제목 (연구 계획서 번호 BMT 1) (Version 2.0)

1. 영문 : Pilot Study for Cardiac Rehabilitation Exercise using a Wearable Device.

국문 : 웨어러블 디바이스 기반 심장재활 운동 예비 연구

2. 시험 책임자

책임연구자 : 노 세 응

3. 개요

본 연구는 사전에 지정된 목표심박수 범위를 바탕으로, 운동중 웨어러블 디바이스를 이용한 실시간 심박측정을 기반으로 적절한 운동강도를 자동으로 추천해주며, 동시에 운동상태에 대해서 알려주는 개발을 위해 활용될 것입니다. 귀하는 건강한 정상 성인 혹은 심장질환자로서 본 연구에 참여하도록 권유를 받았습니다. 귀하가 본 임상연구에 자발적인 의지로 참여하기를 희망 하신다면 동의서에 자필 서명을 하셔야 하며, 대상자 설명서 및 동의서 사본 1부를 받게 됩니다.

이 연구는 자발적으로 참여 의사를 밝히신 분에 한하여 수행 될 것이며, 귀하께서는 본 연구에 참여 의사를 결정하기에 앞서, 본 연구가 왜 수행되고, 귀하의 정보가 어떻게 사용될지, 본 연구가 어떤 것을 포함하고 있는 지와 가능한 이점, 위험, 불편함은 무엇인지에 대하여 이해하는 것이 중요합니다. 또한 귀하께서는 본 연구에 참여를 거부하시면 언제라도 중도에 참여를 포기할 수 있으십니다.

다음의 설명문은 본 연구에 참여할 경우 귀하께서 알아야 할 내용을 자세히 설명해 놓은 것이므로 신중하게 시간을 가지고 주의 깊게 읽으시기 바라며, 만일 어떠한 질문 사항이 있으시면 언제든지 담당자에게 문의하시기 바랍니다.

4. 연구의 목적

이 연구의 목적은 건강한 성인 및 심장질환자에서 사전에 지정된 목표심박수 범위를 바탕으로, 운동중 웨어러블 디바이스를 이용한 실시간 심박측정을 기반으로 적절한 운동강도를 추천해주며, 동시에 운동상태에 대해서 알려주는 개발을 위한 연구입니다.

5. 연구의 방법

(심전도, 심박수 측정 수집 및 제공)

지원자는 본원 게시판 대상자 모집공고를 통해 재활의학과 노세응 교수 (063-859-1622)에게 참여의사를 전하여 참여하게 됩니다.

연구기간은 2015년 1월 17일부터 2016년 12월 31일까지입니다.

지원자는 1회 본원 심장재활치료실에 내원하여 시행합니다. 소요시간은 40분입니다.

1. 귀하의 동의에 하에 성별, 나이, 과거병력, 현병력을 묻고 안정시 심전도, 혈압, 심박동수를 측정하여 정상범위에 있는지 확인하여 측정 진행유무를 판단합니다.
2. <정상인군> - 정상범위로 확인된 지원자분은 홀터 심전도 기기를 장착합니다.

<심장질환자군> - 안정시 심전도, 혈압, 심박동수가 정상범위로 확인되며, 급성기 의학적 치료후 4주이상 경과한 안정상태의 심장질환자 (심근경색, 협심증, 심부전), 최근 6개월내에 운동부하검사를 시행하여 활차운동의 안전한 수행이 가능한 심폐, 근골격 기능을 가진자로 확인된 지원자분은 홀터 심전도 기기를 장착합니다.

(ACSM’s Guideline 기준, 운동부하 검사 의 상대적, 절대적 금기 및 심장재활운동의 금기에 해당하지 않는 환자)

1. 지원자는 안정한 상태로 홀터 심전도 기기로 심전도를 측정함과 동시에 테스트중인 웨어러블 기기를 이용하여 실시간 심박수를 측정합니다.
2. 활차(런닝머신)에서 목표심박수 범위와 현재 심박수를 근거로 속도가 빨라지거나 느려지게 됩니다.
3. 모든 시험군에게 심장의 이상 및 다른 건강의 위험을 초래할 수 있는 증상 및 심전도, 혈압, 맥박의 이상이 있을시 시험을 중단합니다.

6. 대상자에게 예견되는 부작용, 위험과 불편함

<건강한 환자군>- 연구와 관련된 별도의 처치나 약물투여가 없으며 운동강도가 건강한 성인의 일상적인 운동범위를 초과하지 않아 연구 관련 특정 손실 및 위험은 없으나 운동중 부상, 운동후 피로, 잠복된 심장질환 증상발현이 있을 수 있습니다.

<심장질환자군>- 연구와 관련된 별도의 처치나 약물투여가 없으며 6개월내 시행한 운동부하검사 및 의무기록을 확인하여 환자 개인별로 안전하게 수행가능한 강도까지의 운동을 시행하여 연구관련 특정 손실 및 위험은 없으나 운동중 부상, 운동후 피로, 잠복된 심장질환 증상발현, 기존 심장질환의 악화가 있을 수 있습니다.

7. 대상자에게 예견되는 이득

귀하가 본 임상 연구에 참여 함으로서 안정중 심전도 검사, 운동중 심전도 검사를 무료로 시행받게 됩니다.그리고, 이 연구에서 얻은 정보는 심장재활 웨어러블 기기의 개발에 활용되어 심장재활치료를 받는 환자의 치료에 도움이 될 수 있습니다.

8. 동의 철회

귀하는 본 연구의 참여 여부는 자신의 자유의지에 의한 것이어야 합니다.

본 연구의 참여에 동의하였다고 할지라도 시험 중 언제라도 중도에 철회할 수 있습니다. 이 경우 그로 인한 어떠한 불이익도 받지 않습니다. 또한 연구 계속 참여 여부에 영향을 미칠 줄 수 있는 새로운 정보(혈압, 심박수, 심전도 이상, 활차운동이 위험한 의학적 상황발생)를 취득하면 적시에 대상자 또는 대리인에게 알릴 것입니다.

10. 대상자 안전보호에 관한 대책

<건강한 성인군>- 연구와 관련된 특정 약물이나 치료는 운동강도가 건강한 성인의 일상적인 운동범위를 초과하지 않아 연구 관련 특정 손실 및 위험은 없으나 운동중 부상, 운동후 피로, 잠복된 심장질환의 증상발현등에 대해서는 의료진 및 응급처치 장비가 준비된 심장재활치료실에서 이루어 지기 때문에 즉각적인 처치가 가능합니다.

<심장질환자군>- 연구와 관련된 별도의 처치나 약물투여가 없으며 6개월내 시행한 운동부하검사 및 의무기록을 확인하여 환자 개인별로 안전하게 수행가능한 강도까지의 운동을 시행하여 연구관련 특정 손실 및 위험은 없으나 운동중 부상, 운동후 피로, 잠복된 심장질환 증상발현, 기존 심장질환의 악화가 있을 수 있습니다.

11. 윤리적 문제 및 비밀 보장

본 임상연구는 대상자의 보호를 위해 세계의학협회가 발표한 헬싱키선언을 준수하며 수행될 것입니다. 대상자의 신원을 파악할 수 있는 기록은 비밀로 보장될 것이며, 연구의 결과가 출판될 경우 대상자의 신원은 비밀상태로 유지될 것입니다.

12. 개인 정보 보호 및 사용

본 연구를 통해 귀하가 제공하는 개인정보의 범위는 성별, 나이, 병력, 심전도, 혈압, 심박수이며 귀하의 개인정보는 개인식별정보를 익명화 한 후 연구에 사용되며, 항상 엄격하게 기밀 유지될 것입니다. 다만, 점검을 실시하는 자, 기관생명윤리 위원회 위원, 허가관련 당국은 시험절차와 자료의 신뢰성을 검증하기 위한 목적으로 자료를 열람할 수 있습니다. 귀하는 본 동의서에 서명함으로써 본 연구에 해당하는 개인 정보의 이용 및 열람을 허용하게 됩니다.

13. 상담

귀하는 연구 도중 의문사항이 있을 경우 언제든지 시험 담당자인 재활의학과 노세응 교수(063-859-1622)와 상담하거나 추가 정보를 요청할 수 있습니다. 또한 귀하는 연구 대상자로서 안전성 및 윤리적 측면에 이의사항이나 요구 사항이 있을 경우에는 원광대학교 의과대학병원 기관생명윤리위원회(063-859-2232, 2234)로 연락할 수 있습니다.

- - **시험책임자 :** 노세응 재활의학과 교수

[전화] 063-859-1622

- - **모니터기관/담당자:** 원광대학교병원 기관생명윤리위원회

[전화] 063-859-2232

20 년 월 일

대상자 본인 (성명 /자필 서명 ):

20 년 월 일

법정 대리인 (대상자와의 관계 /성명 /자필 서명 ):

20 년 월 일

동의 구득자 (성명 /자필 서명 ):
