# Supplementary material for: Dedicated cardiac rehabilitation wearable sensor and its clinical potential
Source: PLoS One. 2017 Oct 31;12(10):e0187108. doi: 10.1371/journal.pone.0187108 (PMC5663433; doi:10.1371/journal.pone.0187108)
Supplement: S2 Document — The translated statements and written consents in English. (DOCX) [file pone.0187108.s002.docx]

**Statements and Written Consent**

1. Title of the study (research plan number BMT 1) (Version 2.0)

Pilot Study for Cardiac Rehabilitation Exercise using a Wearable Device.

2. Exam officer

Responsible researcher : Se-Eung Noh

3. Overview

This pilot study is to use a wearable device for cardiac rehabilitation exercise. Based on the specified target heart rate range, the real-time measured heart rate information automatically provides a user with an appropriate exercise intensity along with exercise current stage information You are a healthy normal adult or a cardiac disease-related patient, who is recommended to cardiac rehabilitation exercise by cardiac specialists. If you want to be involved in this study voluntarily, you sign at the end of this document. Then, you will get a copy of this statement and written consent.

This study is only for people who want to be involved voluntarily. Before this study, you must know why, and how your test results are used. In addition, you should understand what are beneficial and what are dangerous and discomfort you. More importantly, you should know that you can give up this test at any time.

If you participate in this study, you should carefully take your time to read this statement. if you have any question, please contact a representative at any time.

4. purpose of research

The purpose of this study is for people In healthy adults and heart disease. You will be first tested for measuring target heart rates during exercise. Based on the target heart rates, you wear a watch type wearable device, which estimates real-time heart rate. With the measured heart rates and the target heart rates, you will be informed the exercise intensity. Red LEDs mean you pace up. Yellow LEDs mean you pace down. Green LEDs mean you keep your pace. This is automatic exercise intensity recommendation system. In addition, the wearable device lets you know the exercise stage such as warm-up (yellow LED in the middle), main exercise (green LED in the bottom), cool-down (yellow LED in the middle) and finish (red LED in top). The exercise information can be transmitted to smartphone application. Such a cardiac rehabilitation exercise wearable device will be tested during the exercise on a treadmill.

5. research methods

(Electrocardiogram, heart rate measurement is collected and provided)

Applicants for this recruitment are selected by Professor Se-Eung Noh (063-859-1622) who is a rehabilitation physician.

The research period is January 17, 2015 to December 31, 2016.

Applicant visits the cardiac rehabilitation clinical room. The total study time is 40 minutes.

1) Based on your agreement, we ask your gender, age, past medical history and current medical history, and measure your resting ECG, blood pressure, heart rate. We also evaluate those values are in the normal range and decide to test further.

2) < normal group >- The normal range candidates identified are with ECG devices.

<Heart disease group >- resting ECG, blood pressure, heart rate will be evaluated whether they are in the normal range. Based on ACSM guideline, we carefully make a decision whether you can be tested or not.

3) Applicants measuring ECG device will wear our developed wearable device. The signal measurement will be performed simultaneously.

4) Based on the current heart rate and target heart rates, the speed will be changed on a treadmil.

5) Symptom of heart and any symptom related health problems will be monitored. If any symptom is monitored, the test will be immediately stopped.

6. Expected side-effect, risks and discomfort

<Healthy patients group>- There are no critical side-effects, risks and discomforts. However, you may have injuries during exercise, fatigue after exercise.

<Heart disease group >- There are no critical side-effects, risks and discomforts. However, you may have injuries during exercise, fatigue after exercise. With too strenuous exercise, the heart disease can be worse. Thus, we will monitor your status all the time. You also need to immediately tell us if you feel any uncomfortable.

7. Expected benefits

By participating in this clinical study, you will be provided free ECG test on resting and during exercise. In addition, this tested wearable device will be eventually utilized for cardiac patients.

8. Withdrawal of consent

Your participation in this study is voluntary.

Even if you agree to participate in this study, you can withdraw the agreement at any time during. There is not any disadvantage. In addition, we will inform you if we find any other problem such as blood pressure, heart rate, electrocardiogram,

10. Safety strategy

<Healthy adult group>- There are no critical side-effects, risks and discomforts. However, we will prepare all the emergency equipment. The treatment will be performed immediately.

<Heart disease group >- There are no critical side-effects, risks and discomforts. However, you may have injuries during exercise, fatigue after exercise. With too strenuous exercise, the heart disease can be worse. We will prepare all the emergency equipment. The treatment will be performed immediately.

11. Ethical issues and confidentiality

We protect you based on the World Medical Association Declaration, Helsinki declaration. All the subject information will be strictly confidential. Even after the research results and publication, the all information will be kept secretly.

12. Privacy and use

In this study, the scope of the personal information you provide such as gender, age, medical history, ECG, blood pressure, heart rate will be anonymized. However, the information may be open to the Agency Committee on bioethics for the credibility of the authorities, permission-related test procedures and materials for the purpose of verifying the data. By signing this consent form, you are allowed to check your data.

13. consultation

If you have any questions during your test, you can contact with Professor Se-Eung Noh any time (063-859-1622). In addition, you may ask addition consultation if you need any other information. Also, you can contact with Wonkwang University Medical College Hospital agency Bioethics Committee(063-859-2232, 2234) if you have questions.

◼ **Exam officers :** the Department of rehabilitation [Phone] 063-859-1622

◼ **Monitoring Agency/person :** Wonkwang University Hospital Institutional bioethics committees

                  [Phone] 063-859-2232

Year month day

For the applicant (name /signature               ):

Year month day

The legal representative (relations /name /signature          ):

Year month day

Third party consent (Name /signature                 ):
